# Breastfeeding competency scale (BCS); development and validation of an evaluation instrument on breastfeeding competency in third trimester pregnancy

**DOI:** 10.1186/s12884-021-03664-1

**Published:** 2021-03-04

**Authors:** Yu Wu, Ying Wang, Jiazhen Hu, Yan Dang, Yuanyuan Zhang, Xiumei Qi, Qingxiu Tian, Aihua Wang, Yunfeng Li

**Affiliations:** 1 School of Nursing, Weifang Medical University, 7166 Baotong West Street, Weifang, 261053 Shandong province China; 2 Department of Nursing, Shandong Provincial Qianfoshan Hospital, 16766 Jingshi Street, Jinan, 250014 Shandong province China

**Keywords:** Breastfeeding, Psychometric testing, Scale development, Competency, Validation

## Abstract

**Background:**

Breastfeeding plays an important role in the early stages of humans and throughout the development process. Breastfeeding competency is a self-assessment of pregnant women’s overall competency to breastfeeding which could predict the breastfeeding behaviours of pregnant women. However, a valid and reliable scale for assessing breastfeeding competency has not yet been developed and validated. This study was conducted to develop and validate an assessment scale designed to assess pregnant women’s breastfeeding competency in the third trimester: the Breastfeeding Competency Scale (BCS).

**Methods:**

The BCS was developed and validated over three phases between September 2018 and September 2019, and these phases included item statistical analysis, exploratory factor analysis (EFA), content validation, internal consistency assessment, split-half reliability assessment and confirmatory factor analysis (CFA).

**Results:**

The item statistical analysis and EFA resulted in 38 items and 4 factors that explained 66.489% of the total variance. The Cronbach’s *α* coefficients for the total scale and the 4 factors were 0.970, 0.960, 0.940, 0.822 and 0.931. The split-half reliability of the BCS was 0.894 and 0.890. CFA model showed that the 4-factor model fits the data well.

**Conclusions:**

The BCS is a new valid and reliable instrument for assessing the breastfeeding competency of pregnant women in the third trimester.

**Supplementary Information:**

The online version contains supplementary material available at 10.1186/s12884-021-03664-1.

## Background

Breastfeeding plays a self-evident role in the entire personal process of growth and development. It promotes maternal physical and psychological recovery, contributes to the early initiation of infants’ growth and development, and decreases the risk of long-term disease [1–4]. Promoting and supporting breastfeeding can bring ten times the economic return and a billion-dollar demographic dividend [5, 6]. To that end, large-scale breastfeeding promotion measures have been implemented worldwide [7]. With active protection, promotion, and support, breastfeeding initiation and duration increase. Nevertheless, 78 million babies are not breastfed early within the first hour of life and receive the few benefits they deserve. Therefore, effective breastfeeding intervention studies are still necessary. Some recent studies have also indicated that the rate of exclusive breastfeeding is 38% and the rate of exclusive breastfeeding among 30.71% countries around the world within 6 months of birth is lower than 20% [8–10]. The initiation and duration of breastfeeding behaviours are affected by multiple factors such as individual factors and the socio-economic and healthcare systems [4]. Psychological studies have shown that competency also influences behaviours. Breastfeeding behaviours have been studied through a psychological competency iceberg model to develop and validate a new assessment scale for breastfeeding competency.

In the competency iceberg model, abilities are defined as individual, underlying, and deep-seated features that can distinguish between high and average performers in any jobs. They can be personal features, attitudes and knowledge, cognition or skills in any field that can be reliably measured or counted [11, 12]. Competency is formed, developed and expressed in activities, and it is a prerequisite for engaging in activities. As a kind of competency, breastfeeding competency is defined as the maternal grasp of different breastfeeding factors, such as cognition, motivation, skills and other factors, which intervene in the breastfeeding behaviour based on understanding, controlling and grasping these factors together at the same time. Breastfeeding competency affects the initiation, duration and experience of breastfeeding [13, 14]. Breastfeeding competency can also be prerequisite for establishing and implementing breastfeeding interventions. Therefore, breastfeeding competency requires greater attention.

Some breastfeeding assessment questionnaires have been developed such as the Breastfeeding Knowledge Scale and Breastfeeding Self-Efficacy Energy Scale (BSES) [15, 16]. These questionnaires seem more suitable for assessing breastfeeding behaviour than for assessing breastfeeding competency, while most of them have been assessed to clarified the status quo of breastfeeding self-efficacy or knowledge. Although one questionnaire for assessing breastfeeding competency has been developed, it lacks certain items reflecting breastfeeding knowledge and skill and lacks certain psychometric tests [14]. Furthermore, the “Thirteenth Five-Year Plan” of Health and Wellness Planning proposed vigorously promoting breastfeeding and providing guidance on infant nutrition and feeding [17]. For these reasons, breastfeeding competency should be assessed fully, and doing so will improve the development of breastfeeding support and education. Therefore, our study aims to develop and validate an assessment scale designed to assess the breastfeeding competency.

In 1973, the competency iceberg model was first established by Harvard psychologist McClelland. It is a common model for assessing competency, which includes four part: knowledge, skills, self-concept, traits, and motivation [18, 19]. Knowledge and skills are the visible part of the iceberg above the water, while motivation and traits are the hidden part of the iceberg underwater. Self-concept lies between these two sets of factors and can be changed by education, psychology and accumulated experience in long term [20]. The competency iceberg model has been used in related research on capacity building in different disciplines; for example, Ma Chifen formulated a senior caregiver competency model to promote the development of elderly care [21].

To form a scientific and comprehensive assessment scale for breastfeeding competency, breastfeeding-related content was included in the competency iceberg model. Breastfeeding knowledge is categorized as “knowledge”, breastfeeding skills as “skills”, breastfeeding self-efficacy and maternal self-concept as “self-concept”, and attitudes and social breastfeeding-related support as “ traits and motivation”. Breastfeeding competency can be broken down into different parts that are easier to assess, and the divided parts are closely related and independent of each other, meaning that they can more reliably represent and evaluate breastfeeding competency.

## Methods

### Design

The items of the Breastfeeding Competency Scale (BCS) were designed on the basis of a breastfeeding competency framework which was constructed based on the competency iceberg model, a review of the combined literature published in international or Chinese academic journals and qualitative interviews, with the BSES [16], Breastfeeding Knowledge Scale [15] and Conceptual Model of Components of an Enabling Environment for Breastfeeding [4] taken as references.

#### Qualitative interview

First, qualitative interviews and in-depth interviews with 15 pregnant women were conducted to understand the status and needs of maternal breastfeeding. The literature review was used as the basis of developing the semi-structured interview guidelines. It is the interviewees ranged in age between 25 and 38 years. Half of them were primiparous women, and other half were multiparous women. The qualitative interview results showed that the deficiencies in and needs of maternal breastfeeding included theoretical knowledge, skills, psychological aspects, social support, and the breastfeeding self-concept. Pregnant women required professional guidance related to knowledge and skills, adequate social support, and convenient access to breastfeeding support. They questioned their breastfeeding competency and behaviours because they might not know negative psychological changes due to vague self-concepts.

#### Breastfeeding competency framework

Then, the Delphi method was applied to form a breastfeeding competency framework based on the competency iceberg model and a review the literature published in international or Chinese academic journals. The Delphi method is widely used to reach a consensus among specialists. There Delphi rounds were performed with 14 specialists whose research interests were maternity and midwifery management, education and clinical nursing; 5 of them were international board certified lactation consultants (IBCLCs). The specialists scored the representativeness of each indicator from 0 to 5 (5 is very representative, 0 is not representative). Meanwhile, they commented on issues regarding representativeness and proposed adding, deleting or merging indicators. The mean and coefficient of variation (CV) of each indicator were calculated based on all the specialist responses. If an indicator obtained a mean score less than 3.5 or a CV greater than 25%, it was deleted. After deleting, adding and merging, a breastfeeding competency framework including 3 first-level indicators, 8 s-level indicators, and 54 third-level indicators was formed by the appraised indicators such as the positive coefficients and authority coefficients of the experts, and Kendall’s coefficients of concordance [22].

#### Content validity

Based on the results, after deletion and combination, the BCS, which includes 44 items, was formed through group discussions, and 5 IBCLC specialists who had experience in breastfeeding practice and guidance assessed the validity of the items. A four-point Likert-type response scale was applied in the scale on the preliminary index system of breastfeeding competency, and the scale-level content validity index (S-CVI) and item-level content validity index (I-CVI) were used to assess content validity. The categories of relevance were marked as follows: 1 = “not at all”, 2 = “somewhat”,3 = “moderately so”, and 4 = “very much so” . The I-CVI is calculated as the percentage of experts giving either 3 or 4, and the S-CVI is calculated as the average of the I-CVI of all items. The S-CVI and I-CVI were calculated as 0.965(> 0.9) and 0.875–1.000(> 0.78) respectively. The results indicate that the 44 items of the scale have content validity, and the 44 items were then processed for further psychometric testing.

### Study procedure

In the department of gynaecology and obstetrics, consent forms including the content and methods of this study were distributed. After agreeing to participate, pregnant women completed questionnaires, while standardized explanations of the research objectives, research procedures, and questionnaires were given to the pregnant women to ensure their full understanding. The questionnaires were completed on the day, the participants obtained the questionnaires, and they were sent to the researchers on the same day or during next hospital visit.

### Measures

The data collected consisted data from a questionnaire with socio-demographic characteristics and the BCS.

#### Questionnaire with socio-demographic characteristics

The socio-demographic characteristics of pregnant women were their age, gestational week, parity, education, and occupation as well as the frequency with which they attended a prenatal education programme for pregnant women.

#### Breastfeeding competency scale (BCS)

Each item of the scale assesses the breastfeeding competency currently possessed by pregnant women. The participants responded to all items using a five-point Likert-type scale (from 1 = completely no corresponding to 5 = completely corresponding). The score for the scale was calculated as the sum of the scores for each item, and the mean score was the sum of the items divided by the number of items answered. Higher scores for the sum of the items indicated a greater breastfeeding competency.

### Sample and sample size determination

Pregnant women recruited from two provincial general hospitals in China between September 2018 and September 2019 were invited to participate in the study. The inclusion criteria were being in the third trimester, being able to read and understand the scale explanations, and having no contraindications for breastfeeding. The exclusion criteria were cognitive deficits and/or the inability to independently participate in this study. Regarding the number of women, at least 10–15 times the total number of items should be invited to satisfy the criteria of factor analysis [23]. A total of 440–660 participants were required because the draft BCS included 44 items.

### Data collection

Sample 1 consisted of 580 pregnant women and was conveniently sampled by selecting participants from two provincial general hospitals in China based on the predefined methods and inclusion and exclusion criteria. Completion of the questionnaires (missed items or random answers) was reviewed after the pregnant women completed the questionnaires. This sample was applied for item analysis as well as to assess the reliability and validity of the scale and to construct the BCS.

Sample 2 consisted of 280 pregnant women and was conveniently sampled by selecting participants from two provincial general hospitals in China, with the same methods and criteria as those used with sample 1. Completion of the questionnaires (missed items or random answers) was reviewed. This sample was applied to assess the scale’s effectiveness and degree of data fitting.

### Ethical considerations

This study obtained approval from the Medical Ethics Committee of Weifang Medical College (2019SL060) and consent from the ethics committees of the two hospitals. Informed consent was obtained from the pregnant women and their personal information was kept strictly confidential.

### Data analyses

The socio-demographic characteristics and scale scores of the pregnant women were analysed using descriptive statistics. Means and standard deviations (SDs) were used to calculate the measurement data, such as the age of the pregnant women and the item scores. For the count data, such as the educational background and occupation of the pregnant women, frequencies and percentages were used. The data were analysed using SPSS version 23.0 or AMOS version 23.0. *P* < 0.05 was taken as the level of statistical significance.

#### Item statistics

1. Ceiling effect or floor effect: The percentage of participants who scored the highest and lowest value for each item were obtained and analysed. The items with a ceiling effect (where the percentage of participants was more than 15%) or a floor effect (where the percentage of participants was more than 15%) were considered for deletion [24].
2. Item distribution method: The distribution of item options was obtained and analysed. If the selection rate of each option for an item accounted for more than 80%, the item was considered for deletion [25].
3. The coefficient of variation method: The means and SDs of each item were calculated, and the CV was calculated as follows: CV = s/x. Items with a low CV (< 15%) were considered for deletion.
4. The critical ratio (CR) analysis method: Based on the total score of the scale, the top 27% was regarded as the upper group, and the bottom 27% was regarded as the lower group. The two groups were tested by an independent-samples t test [26]. Items were deleted when *P* > 0.05.
5. The correlation coefficient method: Pearson’s correlation coefficient was used to evaluate the scores of each item and the total score of the scale. Items with a low coefficient (< 0.2) and/or a high *P* value (> 0.05) were considered for deletion.
6. The factor analysis method: After factor analysis, item with a cumulative variance contribution rate of common factors < 40%, factor loading values< 50%, or factor loadings on a non-unique common factor > 50% were considered for deletion [27].
7. Cronbach’s α coefficient method: Cronbach’s *α* coefficient was used to analyse the internal consistency of the scale, and if the existence of an item caused the Cronbach’s *α* coefficient to decrease, the item was considered for deletion [28].

Following these methods, items were deleted when they met more than three criteria. Moreover, if an item met less than three criteria, it was discussed for modification, merging or deletion based on the group discussions with experts.

#### Reliability

Cronbach’s α coefficient and split-half reliability were used to test the reliability of the scale. For the total scale and the factors and items, Cronbach’s *α* coefficient ≥ 0.7 and Spearman-Brown split-half reliability and Guttman split-half reliability ≥0.7 were assessed as fitting [25, 29].

#### Validity

Content validity, Pearson’s correlation analysis, exploratory factor analysis (EFA) and confirmatory factor analysis (CFA) were used to test the validity of the scale. First, the content validity of the scale was tested by the Delphi method. Second, the correlation coefficient analysis was performed among the factors and between factors I-IV and the BCS. Third, EFA and CFA were carried out based on a two-step strategy. Sample 1 was randomly selected for EFA, and sample 2 was selected for CFA to verify the fitting degree of the model. The validity of the scale is trustworthy when the following criteria are confirmed. In the content validity method, I-CVI > 0.78 and S-CVI/Ave > 0.9 are acceptable [30]. The range of the correlation coefficients among the factors is 0.1–0.6, and the correlation coefficients between factors I-IV and the BCS are 0.3–0.8. In EFA, there should not be less than 3 items under each common factor [26]. In CFA, the model should meet the following criteria [31].

## Results

A total of 580 pregnant women in the third trimester were enrolled in the first round and 565 (97.41%) valid questionnaires were completed. A total of 280 pregnant women were enrolled in for the second round and 244 (87.14%) valid questionnaires were completed and obtained.

### Item analysis

#### Item statistics

The mean score for each BCS item varied between 2.989 and 4.520, with SDs ranging between 0.705 and 1.118 and CVs ranging between 0.16 and 0.37. The range of all item options from 1 = completely no corresponding to 5 = completely corresponding was 0.708–9.381%, 0.177–27.434%, 2.832–50.796%, 15.221–49.735%, and 9.204–61.593%. No items had a floor effect, some items had ceiling effects, and the distribution of all items’ options was less than 80%. The CVs of the items were between 0.16 and 0.37, and in the CR, all items were statistically significant (*P* < 0.05) except item 6. The correlation coefficient between the items and the BCS score were between 0.366 and 0.805, and for item deletion, the Cronbach ‘s α coefficients were between 0.967 and 0.969, with the exception of item 6 (see Table 1).

**Table 1:** Item statistics results of the draft Breastfeeding Competency Scale (44 items, *n* = 565)

Item	Coefficient of Variation	critical ration t	Cronbach’s alpha if item deleted	correlation coefficient
	Mean (SD)			
1	3.773 (0.935)	16.576^**^	0.968	0.642^**^
2	4.004 (0.936)	18.361^**^	0.968	0.673^**^
3	3.968 (1.021)	23.117^**^	0.967	0.769^**^
4	4.027 (0.933)	14.742^**^	0.968	0.630^**^
5	3.641 (1.021)	15.252^**^	0.968	0.621^**^
6	3.030 (1.118)	−0.244	0.971	−0.057
7	3.781 (1.029)	7.722^**^	0.969	0.366^**^
8	3.581 (1.070)	21.340^**^	0.968	0.728^**^
9	4.520 (0.757)	8.124^**^	0.969	0.388^**^
10	3.501 (0.948)	20.439^**^	0.967	0.761^**^
11	3.481 (0.916)	21.720^**^	0.967	0.790^**^
12	3.738 (1.031)	24.655^**^	0.967	0.778^**^
13	3.729 (1.026)	23.030^**^	0.967	0.766^**^
14	3.855 (1.035)	21.554^**^	0.968	0.741^**^
15	3.991 (0.894)	17.734^**^	0.968	0.727^**^
16	3.807 (0.899)	20.049^**^	0.968	0.751^**^
17	3.480 (0.996)	25.485^**^	0.967	0.805^**^
18	4.297 (0.705)	13.685^**^	0.968	0.553^**^
19	3.843 (0.979)	17.867^**^	0.968	0.697^**^
20	3.255 (0.941)	12.033^**^	0.968	0.570^**^
21	3.292 (0.959)	20.256^**^	0.967	0.792^**^
22	3.818 (0.834)	19.796^**^	0.968	0.729^**^
23	3.478 (0.933)	20.321^**^	0.967	0.761^**^
24	3.377 (0.944)	18.901^**^	0.968	0.728^**^
25	3.662 (0.931)	21.979^**^	0.968	0.748^**^
26	3.524 (0.997)	20.409^**^	0.968	0.739^**^
27	3.020 (0.961)	14.615^**^	0.968	0.649^**^
28	3.304 (0.964)	19.381^**^	0.968	0.726^**^
29	3.432 (1.023)	19.831^**^	0.968	0.740^**^
30	3.542 (0.953)	19.284^**^	0.968	0.710^**^
31	3.363 (1.013)	15.592^**^	0.968	0.635^**^
32	2.989 (1.050)	8.153^**^	0.969	0.442^**^
33	3.570 (0.995)	19.029^**^	0.968	0.716^**^
34	3.901 (0.913)	20.069^**^	0.968	0.745^**^
35	3.805 (0.872)	24.512^**^	0.967	0.788^**^
36	4.119 (0.764)	17.073^**^	0.968	0.662^**^
37	3.795 (0.979)	22.638^**^	0.967	0.754^**^
38	3.361 (1.052)	13.999^**^	0.968	0.606^**^
39	4.108 (0.795)	15.530^**^	0.968	0.638^**^
40	4.243 (0.805)	12.757^**^	0.968	0.580^**^
41	3.936 (1.012)	11.438^**^	0.969	0.492^**^
42	4.331 (0.764)	12.409^**^	0.968	0.510^**^
43	3.300 (1.021)	20.912^**^	0.967	0.775^**^
44	4.205 (0.853)	10.717^**^	0.969	0.490^**^

Note.^*^P < 0.05, ^**^P < 0.001

*SD* Standard Deviation

#### Factor analysis

A total of 3 rounds of factor analysis were performed, and 4 factors with eigenvalues >1.0 were identified. After 6 items were deleted, the factor loadings of all items were showed good psychometric properties. The excluded items included “6 I would not use pacifier during lactation”, “7 I would not wear breast-patches or bras during lactation”, “9 I would avoid bad habits during lactation”, “18 I would keep my breasts clean and dry”, “19 I would ask galactagogue division for help for breast pain”, and “35 I know how to strengthen my attachment relationship with baby”.

### Factor structure

**Fig. 1:**
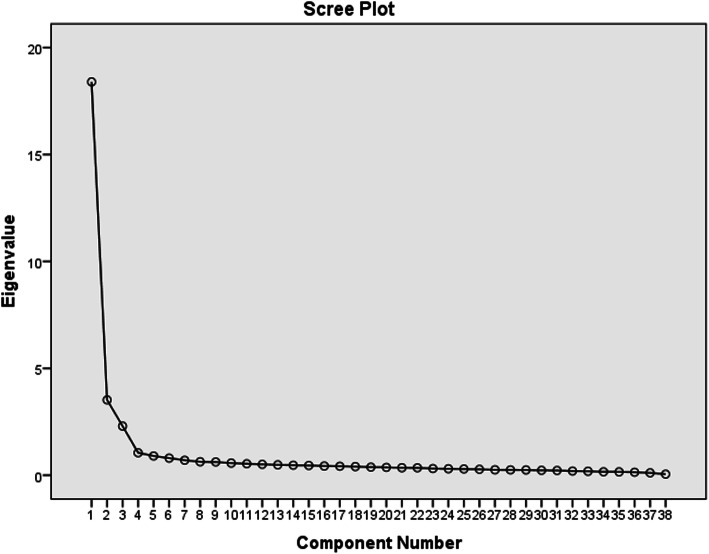
Scree plot showing cut-off point for scale factors (*n* = 565)

The Kaiser-Meyer-Olkin (KMO) coefficient of this sample was 0.968 and the approximate chi-square value of the Bartlett’s test was 18,525.221 (*P* < 0.05), which indicated that the sample was suitable for EFA. The scree plot showed a curve that levelled off at factor 4 with a corresponding eigenvalue > 1 (see Fig. 1). Four factors were identified and explained 66.489% of the total variance. Factor 1 contained 15 items and was named “breastfeeding knowledge” with factor loadings between 0.599 and 0.818. Factor 2 contained 11 items and was named “self-concept and psychology” with factor loadings between 0.649 and 0.807. Factor 3 contained 4 items and was named “management of breast-milk” with factor loadings between 0.558 and 0.764. Factor 4 contained 8 items and was named “breastfeeding skill” with factor loadings between 0.586 and 0.774 (see Table 2). The correlations between the BCS (38 items) and the four factors are as follows: r_1_ (BCS and factor 1) = 0.913, r_2_(BCS and factor 2) = 0.874, r_3_(BCS and factor 3) = 0.771 and r_4_(BCS and factor 4) = 0.799 (*P* < 0.001).

**Table 2:** Rotated component matrixa results in exploratory factor analysis for BCS (*n* = 565)

Item	Factor 1 Breastfeeding Knowledge	Factor 2 Self-concept and Psychology	Factor 3 Management of Breast-milk	Factor 4 Breastfeeding Skill
1	0.624			
2	0.732			
3	0.760			
4	0.618			
5	0.637			
8	0.732			
10	0.712			
11	0.694			
12	0.818			
13	0.817			
14	0.794			
15	0.695			
16	0.724			
17	0.673			
20		0.667		
21				0.665
22				0.635
23				0.737
24				0.774
25				0.659
26		0.720		
27				0.595
28				0.586
29				0.613
30			0.664	
31			0.764	
32			0.558	
33			0.626	
34		0.694		
36		0.650		
37		0.655		
38		0.649		
39		0.728		
40		0.807		
41		0.759		
42		0.754		
43	.599			
44		0.748		

### Reliability

The Cronbach’s *α* coefficient for the BCS was 0.970, and the coefficients for the four factors were 0.960, 0.940, 0.822 and 0.931. The Spearman-Brown split-half reliability of the scale was 0.894, and the Guttman split-half reliability was 0.890. These results show that the scale has reliability.

### Validity

**Fig. 2:**
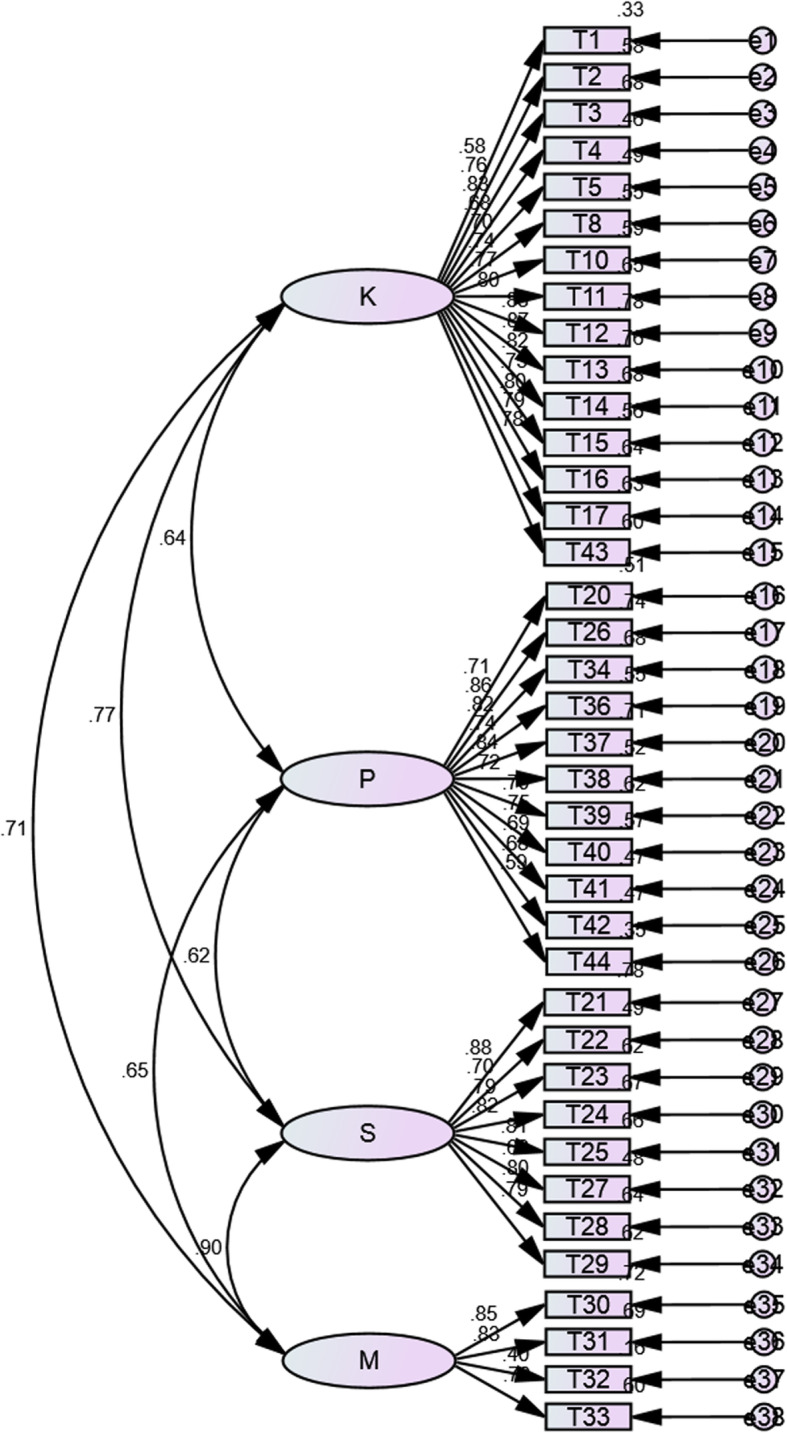
CFA standardised item loadings and factor correlations for Breastfeeding Competency Scale (BCS; *n* = 244; *p* < 0.001)

The four-factor model identified in the factor structure was tested in sample 2 using confirmatory factor analysis to determine validity and appropriateness. The fit indices indicated that the 4-factor model fit the data well (*χ2* = 898.152 (*P* > 0.05), *χ2/df* = 1.363, RMSEA = 0.039) except for the GFI, RFI, NFI, and AGFI (see Table 3). Some indices (GFI = 0.838, AGFI = 0.817, NFI = 0.879, RFI = 0.871) did not align with the expected study results, but they were close to the cut-off values, meaning that they were acceptable. The RMSEA aligns with expectations and is considered to be an appropriate index [26] (see Table 3 and Fig. 2).

**Table 3:** Appropriate indices of model for CFA (*n* = 224)

Absolute Fit Indexs	result	Incremental Fit Indexs	result	Simplicial Fit Indexs	result
*χ*^2^	898.152	NFI	0.879	*χ*^*2*^*/df*	1.363
RMR	0.043	RFI	0.871	PGFI	0.745
RMSEA	0.039	IFI	0.965	PNFI	0.824
GFI	0.838	NNFI/TLI	0.962	PCFI	0.904
AGFI	0.817	CFI	0.964	CN	202

Note. *χ*^2^ Chi-square goodness of fit statistic, *RMR* Square root mean residual, *RMSEA* Root-mean-square Error of Approximation, *GFI* Goodness-of-fit Index, *AGFI* Adjusted goodness-of-fit index, *NFI* Normed Fit Index, *RFI* Relative fit index, *IFI* Incremental fit index, *TLI* Tucker Lewis Index, *CFI* Comparative Fit Index, *df* Degrees of freedom, *PGFI* Parsimonious goodness-of-fit Index, *PNFI* Parsimonious normed Fit Index, *PCFI* Parsimonious comparative Fit Index, *CN* Critical N

## Discussion

The purpose of this study is to construct and evaluate the BCS based on the competency iceberg model. The results show that the BCS with 38 items and 4 factors achieved satisfactory reliability and validity, meaning that it can be used to evaluate the breastfeeding competency of pregnant women.

Factors such as individual factors and the socioeconomic and healthcare systems have been proven to affect the initiation, experience and duration of breastfeeding behaviours [4]. Individual factors including maternal attitudes, self-efficacy, knowledge and peer support, are the reasons for breastfeeding and can predict the initiation and duration of breastfeeding. A review of the literature reported that high-risk pregnancies, mother-infant separation and long hospital stays can cause a later start for breastfeeding and the overuse of breastmilk substitutes. In these cases, women have less self-efficacy and worse attitudes towards breastfeeding than healthy pregnant women, and they erroneously believe that they have low-quality breastmilk [32]. Pregnant women whose peers provide them with the wrong knowledge and skills, for example, with regard to harmful colostrum and non-nutritive mature milk, are more likely to give up breastfeeding [33]. At the socio-economic and cultural level, the duration and experience of breastfeeding are affected by factors including the social environment and economic background. Over-avoidance behaviour and expressions of discomfort by some employers, colleagues and other people will increase pregnant women’s sense of shame. Thus, pregnant women will have negative reactions to breastfeeding, leading to the premature termination of breastfeeding [34]. A short maternity leave and high-intensity work can make pregnant women wean early [35]. In health systems, breastfeeding legislation and policy influence the initiation and duration of breastfeeding by providing the support of healthcare providers. Substantial gaps in breastfeeding knowledge and skills are reported at all levels of healthcare staff [4]. Some healthcare providers providing low-quality breastfeeding support can lead to early weaning. In addition, the initiation of breastfeeding can be put off by breastfeeding alternatives offered by some hospitals [36]. Therefore, at the beginning of this study, these factors were taken into account in the scale design stage.

Previous studies have designed different scales to predict breastfeeding behaviour based on different aspects, and they mainly assess the aspects of breastfeeding psychology and support systems. Different scales contain some common factors or items because of the same predictive content. There are differences in the scope of the scale or the assessment population due to different theoretical frameworks. Various scales have been developed to assess breastfeeding effectiveness, breastfeeding attitudes, and social support. However, it is still necessary to form scales to assess breastfeeding competency. To ensure comprehensiveness, a qualitative study of lactating women and Delphi method research were carried out on the basis of the competency iceberg model. Strict psychological measurements were performed on the scale, abiding by the scientific process.

The BCS was developed based on breastfeeding behaviour and competency, and it is different from previous scales developed based on the effectiveness of breastfeeding behaviour or self-efficacy [15, 16]. The BCS is more suitable than other scales for assessing pregnant women’s breastfeeding competency in the third trimester. On the one hand, both prenatal and postnatal education are important as the incidence of breastfeeding is primarily affected by prenatal education which is usually carried out in the third trimester and the content including breastfeeding knowledge, skills and attitude, is the same as the 4 dimensions of the BCS. Therefore, the dimensions of the BCS are suitable for assessing the breastfeeding competency of pregnant women in the third trimester. On the other hand, competency is formed, developed and expressed in activities such as education, past behaviour or experience. All kinds of pregnant women such as primiparous women and multiparous women, can develop their breastfeeding competency through prenatal breastfeeding education, peer experience sharing and other ways [33]. Therefore, some items of the BCS are developed from the maternal experience of breastfeeding. Pregnant women can perform self-assessments with the BCS to determine whether they can breastfeed with their own breastfeeding competency. The breastfeeding competency of pregnant women in third trimester pregnancy can be assessed, the level of various parts can be understood, and weaknesses can be accurately located the using the BCS.

The BCS showed high and effective response rate during data collection, indicating that the items should be easily understood. The results also indicated that pregnant women had a high degree of interest in this research. Previous research has found that pregnant women might obtain incorrect breastfeeding knowledge, skills, and cognition due to errors and false information from the internet [37]. As a result, pregnant women tended to choose “fit” when completing the BCS. The ceiling effect and floor effect should not appear in instrument development studies, but in this study, the ceiling effect was unavoidable [38]. Some items had a ceiling effect due to pregnant women’s incorrect concept or misunderstanding of breastfeeding competency. However, the selection rate of each option for the items was < 80%, indicating that all items of the BCS have the ability to distinguish between high and average breastfeeding competency [25].

Factor analysis showed that the BCS is a multidimensional assessment scale composed of 4 factors, which was the same as the results of the qualitative study and Delphi method analysis of breastfeeding competency [22]. The four factors include “breastfeeding knowledge”, “breastfeeding skill”, “management of breast-milk” and “self-concept and psychology”. In the above results, “breastfeeding skill” focuses on the skills related to the women or the new-born during the breastfeeding process, while the “management of breast-milk” focuses on the skills related to and the management of extra breast milk before and after the breastfeeding process, which is different from “breastfeeding skill”. Therefore, the results of the factor structure in this study show that “management of breast milk” is separate from “breastfeeding skill” and is an independent factor.

The internal consistency and split-half reliability of the BCS and its 4 factors meet the criteria for psychological instrument development, indicating that the BCS has high reliability and can be considered a good assessment scale [29].

Although quality control was strictly applied throughout the whole study process, certain limitations need to be considered. The first limitation of this study concerns geographical differences. Breastfeeding rates and behaviours are different based on geography, race and culture [2]. Therefore, the participants might not be representative of the whole Chinese population of pregnant women, and they may represent only the situation of the two provincial general hospitals selected in Jinan, Shandong, China. Usually, a large number of pregnant women at different risk levels make regular hospital visits at provincial general hospitals. Therefore, the researchers limited the participants to provincial general hospitals rather than community hospitals. The second limitation of this study is that competency is a dynamic process. A sufficient amount of data could not be collected in a short period of time. However, the third trimester of pregnancy is a relatively stable stage of breastfeeding competency where pregnant women have been exposed to a certain amount of breastfeeding-related knowledge. The choice of the third trimester of pregnancy as an inclusion criterion could mitigate the bias caused by the dynamic process of breastfeeding competency. In future studies, the number of participants should be expanded to evaluate the reliability and validity of the BCS.

## Conclusions

Assessing breastfeeding competency is a key component for developing appropriate breastfeeding support and education. This study developed the BCS theoretically based on the competency iceberg model to evaluate the breastfeeding capacity of pregnant women in the third trimester. The BCS, which includs 38 items showed trustworthy psychometric properties and can be applied as a supplementary assessment scale to predict breastfeeding behaviors. The BCS can also serve as a reference or as an assessment scale for developing early interventions related to pregnant women’s breastfeeding competency to promote effective breastfeeding behaviors and ensure earlier and longer breastfeeding. Therefore, the BCS is a valuable evaluation instrument that can be used as a foundation for midwives and other healthcare professionals with benefits for maternal and child health providers to develop appropriate breastfeeding support and education to meet the individual breastfeeding requirements of pregnant women.

## Supplementary Information

**Additional file 1.** The source of BCS items.

## Data Availability

The data analyzed in this study are not publicly available due to privacy policy, but are available from the corresponding author on reasonable request. For further information about data access, please contact to the corresponding author, Aihua Wang by email.

## Abbreviations

BCS: Breastfeeding Competency Scale
BSES: Breastfeeding Self-Efficacy Energy Scale
IBCLC: International board certified lactation consultant
SD: Standard deviations
CV: Coefficient of variation
CR: Critical ratio
KMO: Kaiser-Meyer-Olkin
EFA: Exploratory Factor Analysis
*χ2*: Chi-square goodness of fit statistic
RMR: Square root mean residual
RMSEA: Root-mean-square Error of Approximation
GFI: Goodness-of-fit Index
AGFI: Adjusted goodness-of-fit index
NFI: Normed Fit Index
RFI: Relative fit index
IFI: Incremental fit index
TLI: Tucker Lewis Index
CFI: Comparative Fit Index
*df*: Degrees of freedom
PGFI: Parsimonious goodness-of-fit Index
PNFI: Parsimonious normed Fit Index
PCFI: Parsimonious comparative Fit Index
CN: Critical N
