# Supplementary material for: Breastfeeding competency scale (BCS); development and validation of an evaluation instrument on breastfeeding competency in third trimester pregnancy
Source: BMC Pregnancy Childbirth. 2021 Mar 4;21:179. doi: 10.1186/s12884-021-03664-1 (PMC7934416; doi:10.1186/s12884-021-03664-1)
Supplement: Supplementary file 1 — Additional file 1. The source of BCS items. [file 12884_2021_3664_MOESM1_ESM.docx]

**Supplementary material files 1** The source of BCS items

| Item | Conceptual Model^[14]^ | Breastfeeding Knowledge scale | BSES | Qualitative  interview | Delphi survey | Literature |
| --- | --- | --- | --- | --- | --- | --- |
| 1.I know the commencement and termination time of breastfeeding. |  | **√** |  | **√** | **√** |  |
| 2.I could make skin-to-skin contact with my baby while breastfeeding. |  |  |  |  | **√** | **√** |
| 3.I know the benefits of breastfeeding. | **√** | **√** | **√** | **√** | **√** | **√** |
| 4.I could be responsive to baby while breastfeeding. |  |  |  | **√** | **√** | **√** |
| 5.I know the merit and demerit of formula as a supplement. |  |  | **√** | **√** | **√** |  |
| 8.I know the precaution and contraindication of drug taking during lactation. |  |  |  | **√** | **√** |  |
| 10.I could identify when my baby is hungry. | **√** |  | **√** | **√** | **√** |  |
| 11.I could identify when my baby is full. |  |  | **√** |  | **√** |  |
| 12.I am concerned with baby’s defecation. (frequency,nature) |  |  | **√** | **√** | **√** |  |
| 13.I am concerned with baby’s urine.(frequency, color) |  | **√** | **√** |  | **√** |  |
| 14.I could observe baby’s growth and development on regular basis. |  |  |  | **√** | **√** |  |
| 15.I know the commencement time of complementary foods for baby. |  |  |  | **√** | **√** |  |
| 16.I know the time when the baby is weaned |  |  |  |  | **√** |  |
| 17.I know the way to wean for baby. |  |  |  |  | **√** |  |
| 20.I could relieve anxiety and depression during lactation. | **√** |  |  |  | **√** |  |
| 21.I know how to promote breast-milk secretion. |  | **√** | **√** |  | **√** | **√** |
| 22.I could breastfeed with comfortable positions. |  |  | **√** | **√** | **√** | **√** |
| 23.I know the way to comfort irritability baby when breastfeeding. | **√** |  | **√** |  | **√** |  |
| 24.I know the way to wake baby up while breastfeeding. |  |  |  |  | **√** | **√** |
| 25.I could guide the baby latching on while breastfeeding. | **√** | **√** | **√** |  | **√** |  |
| 26.I am confident to take breastfeeding during night. |  |  |  | **√** | **√** |  |
| 27.I could control the speed of breast-milk secretion. |  |  |  |  | **√** | **√** |
| 28.I could correct baby nipple confusion. |  |  |  |  | **√** | **√** |
| 29.I know the way to pull nipple out of baby's mouth. |  |  |  | **√** | **√** |  |
| 30.I know the way to store breast-milk. |  |  |  | **√** | **√** | **√** |
| 31.I know the way to reheat frozen or refrigerated breast-milk. |  |  |  |  | **√** | **√** |
| 32.I could milk extra breast-milk by hand. |  | **√** |  |  | **√** | **√** |
| 33.I know the way to use a breast pump. |  | **√** |  | **√** | **√** | **√** |
| 34.I am confident to complete breastfeeding. | **√** |  | **√** | **√** | **√** | **√** |
| 36.I could proactively acquire of knowledge about breastfeeding. |  |  |  | **√** | **√** | **√** |
| 37.I know the pathway to get knowledge about breastfeeding. | **√** |  |  | **√** | **√** |  |
| 38.I could insist on breastfeeding in public areas. | **√** |  |  |  | **√** | **√** |
| 39.I could get support from my family. | **√** |  | **√** |  | **√** |  |
| 40.I think breastfeeding is my responsibility. | **√** |  | **√** | **√** | **√** |  |
| 41.I could not temporarily give up breastfeeding for self-image. |  |  |  | **√** | **√** | **√** |
| 42.I think breastfeeding is important. |  |  | **√** | **√** | **√** |  |
| 43.I know the secretion mechanism of breast-milk. |  |  |  |  | **√** | **√** |
| 44.I think breastfeeding is my obligation. |  |  |  | **√** | **√** |  |
